# Comparison of the association and discriminatory ability of CVAI, LAP, CI, and AVI for type 2 diabetes in Chinese adults aged ≥ 50 years: a sex-specific analysis

**DOI:** 10.3389/fendo.2026.1829222

**Published:** 2026-05-20

**Authors:** Liu Zhang, Liying Wen, Guimei Chen, Jingwen Puyang, Zizhou Cheng, Yu Zhu, Weiwei Chang

**Affiliations:** 1 Department of Hospital Infection Management Office, Wuhu Hospital of Traditional Chinese Medicine, Wuhu, Anhui, China; 2 Department of Epidemiology and Health Statistics, School of Public Health, Wannan Medical University, Wuhu, Anhui, China; 3 School of Health Management, Anhui Medical University, Hefei, Anhui, China; 4 Science and Education Department, Wuhu Hospital of Traditional Chinese Medicine, Wuhu, Anhui, China

**Keywords:** AVI, case-control study, CI, comparative analysis, CVAI, LAP, sex-specific analysis, type 2 diabetes mellitus

## Abstract

**Objective:**

The aim of this study was to compare the associations of four visceral adiposity indices—Chinese visceral adiposity index (CVAI), lipid accumulation product (LAP), conicity index (CI), and abdominal volume index (AVI)—with prevalent type 2 diabetes mellitus (T2DM) in Chinese adults aged ≥ 50 years and to evaluate their discriminatory ability within the study sample. We further investigated whether sex modifies these associations and discriminatory ability.

**Methods:**

In this community-based case-control study with frequency matching by sex and age conducted in Anhui Province, China (1, 088 participants; 491 T2DM cases, 597 controls), we assessed associations using unconditional multivariable logistic regression (fully adjusted model) and discriminatory ability using receiver operating characteristic (ROC) curve analysis, with prespecified sex-stratified analyses.

**Results:**

After full adjustment, all four indices were positively associated with prevalent T2DM. CVAI and CI showed significant associations across Q2-Q4, while LAP and AVI were significant only in Q3-Q4. In terms of discriminatory ability, CVAI had the numerically highest AUC in the overall population (0.634), followed by LAP (0.633), AVI (0.612), and CI (0.591). Sex differences were also observed. CVAI, CI, and AVI showed stronger associations in males, while LAP showed a stronger association in females. Discriminatory ability followed the same pattern, with CVAI showing the numerically highest area under the curve (AUC) in males (0.658) and LAP in females (0.642).

**Conclusion:**

This study suggests that CVAI, LAP, CI, and AVI are independently associated with prevalent T2DM in adults aged ≥50 years, with significant sex modification in both association and discriminatory ability within the study sample. Findings suggest sex-specific differences: CVAI had stronger associations and higher discriminatory ability in males, whereas LAP had stronger associations and higher discriminatory ability in females. As a case-control study from a single province, these findings do not establish causality or predictive utility. Prospective studies are needed to validate these findings.

## 1 Introduction

With global population aging and profound lifestyle transitions, diabetes mellitus (DM) has become a major global public health burden, affecting 529 million adults worldwide (prevalence 6.1%) and projected to reach 1.31 billion by 2050 (1). In China, adult diabetes prevalence rose from 10.9% in 2013 to 12.4% in 2018 (2), with 140 million affected individuals in 2021 (3). The epidemic imposes severe mortality, complications and socioeconomic burdens, with global diabetes healthcare spending exceeding one trillion dollars (4). As type 2 diabetes mellitus (T2DM) accounts for >90% of all diabetes cases and frequently progresses to retinopathy, nephropathy, and cardiovascular complications (5), effective early risk evaluation is needed, especially among adults aged ≥50 years—a group with unique fat distribution patterns and a high T2DM burden (6).

Obesity is a well-established modifiable risk factor for T2DM (7). Although the American Diabetes Association (ADA) integrated body mass index (BMI) and conventional anthropometric metrics into clinical guidelines as standard assessment tools, accumulating evidence regarding adipose tissue heterogeneity has challenged their applicability (8). BMI alone is insufficient to characterize obesity-related phenotypic features; instead, indicators reflecting fat distribution and central adiposity, including waist circumference (WC), waist-to-hip ratio (WHR) and waist-to-height ratio (WHtR), are recommended for diabetes glucose metabolism risk evaluation (8). However, considerable uncertainty remains regarding whether these conventional indices maintain reliable discriminatory ability in older populations, who undergo extensive physiological remodeling associated with aging.

Sex is a critical effect modifier in the obesity–T2DM association, and sex heterogeneity substantially impairs the discriminative utility of anthropometric measurements (9). Aging drives visceral and intermuscular fat infiltration, progressive muscle mass loss, persistent low-grade inflammation and aggravated insulin resistance (10). Evidence indicates that middle age, particularly around 50 years old, constitutes a critical turning point for metabolic dysregulation (11), marking the point where conventional anthropometric indices begin to lose discriminative validity (12). Women possess a higher overall body fat percentage and distinct regional fat deposition patterns, resulting in divergent metabolic risk profiles between males and females (13). A recent Mendelian randomization study verified sex-specific causal associations between body composition and T2DM risk, revealing that gluteofemoral fat exerts independent protective effects against diabetes exclusively in women (14). These findings highlight the limitations of simple circumference-based indices for prevalent T2DM identification in older adults.

To address these limitations, four adiposity indices were selected in this study, covering two complementary biological dimensions: metabolic dysregulation and morphological abdominal fat accumulation (15). The Chinese visceral adiposity index (CVAI) and lipid accumulation product (LAP) reflect internal metabolic burden, whereas abdominal volume index (AVI) and conicity index (CI) capture external abdominal fat distribution independent of lipid profiles. This combined selection integrates both dyslipidemia-associated metabolic disturbance and abdominal adiposity risk, compensating for inherent limitations of single-dimensional assessment in older populations. The 2023 European Association for the Study of Diabetes (EASD) and European Geriatric Medicine Society (EuGMS) joint consensus recognized that metabolic composite indices including LAP and CVAI better reflect visceral fat accumulation, insulin resistance and muscle-fat imbalance in older adults (16). While AVI and CI have not obtained official recommendation, they have been frequently adopted as optional research indicators in comparative adiposity studies (17, 18). Furthermore, statin treatment often normalizes circulating lipid profiles in elderly patients despite persistent central obesity, potentially masking residual metabolic risk; in such cases, lipid-dependent morphological indices may retain greater utility (19).

Each index has been validated in prior clinical research. CVAI, developed specifically for Asian populations, has been consistently associated with T2DM risk and shows discriminative value across multiple cohorts (20–22). LAP, constructed from WC and triglyceride levels, reflects systemic lipid accumulation and is closely correlated with insulin resistance, metabolic syndrome, and T2DM (23, 24). AVI reliably quantifies abdominal fat burden and correlates with metabolic disease susceptibility (25). CI is sensitive to dynamic changes in abdominal fat distribution and uniquely characterizes abdominal adiposity in both obese normal-weight individuals (26, 27). Despite these advances, controversies and knowledge gaps remain. First, no consensus has been established regarding the optimal index, particularly among elderly individuals. Second, findings on the association between CI and T2DM are inconsistent across populations (28–30). Whether CVAI shows higher discrimination than LAP in T2DM remains debatable, with insufficient evidence among older Chinese adults. Third, sex-stratified evidence remains scarce, and direct head-to-head comparative analyses are limited. Further investigation focusing in Chinese adults aged ≥50 years is lacking, despite distinct sex differences in fat distribution, hormonal environment and insulin resistance pathways (24, 31).

Accordingly, this case-control study was conducted to compare the strength of association between each of the four indices (CVAI, LAP, CI, AVI) and prevalent T2DM, and to evaluate their discriminatory ability. The study further investigated whether sex modifies these associations and discriminatory ability, aiming to provide a sex-stratified characterization of these relationships and to generate hypotheses for future longitudinal research.

## 2 Materials and methods

### 2.1 Study design and population

This case-control study was nested within a community-based health examination program in Anhui Province, China, conducted between February 1 and February 28, 2020. This study was reported in accordance with the STROBE statement for case-control studies. Participants were eligible if they were aged ≥50 years and provided written informed consent. Exclusion criteria included type 1 diabetes mellitus, latent autoimmune diabetes in adults (LADA), secondary diabetes (e.g., drug-induced or pancreatic diseases), severe hepatic or renal dysfunction, malignant tumors, or mental disorders that could affect cooperation.

T2DM was defined as meeting at least one of the following: (1) FPG ≥7.0 mmol/L; (2) self-reported physician diagnosis; (3) current use of glucose-lowering medications. Controls were individuals without T2DM (fasting plasma glucose <6.1 mmol/L and no self-reported diabetes or glucose-lowering medication use). Controls were frequency-matched to cases by sex and age in 5-year bands. Sex and age were subsequently included as covariates in multivariable models.

**Figure 1:**
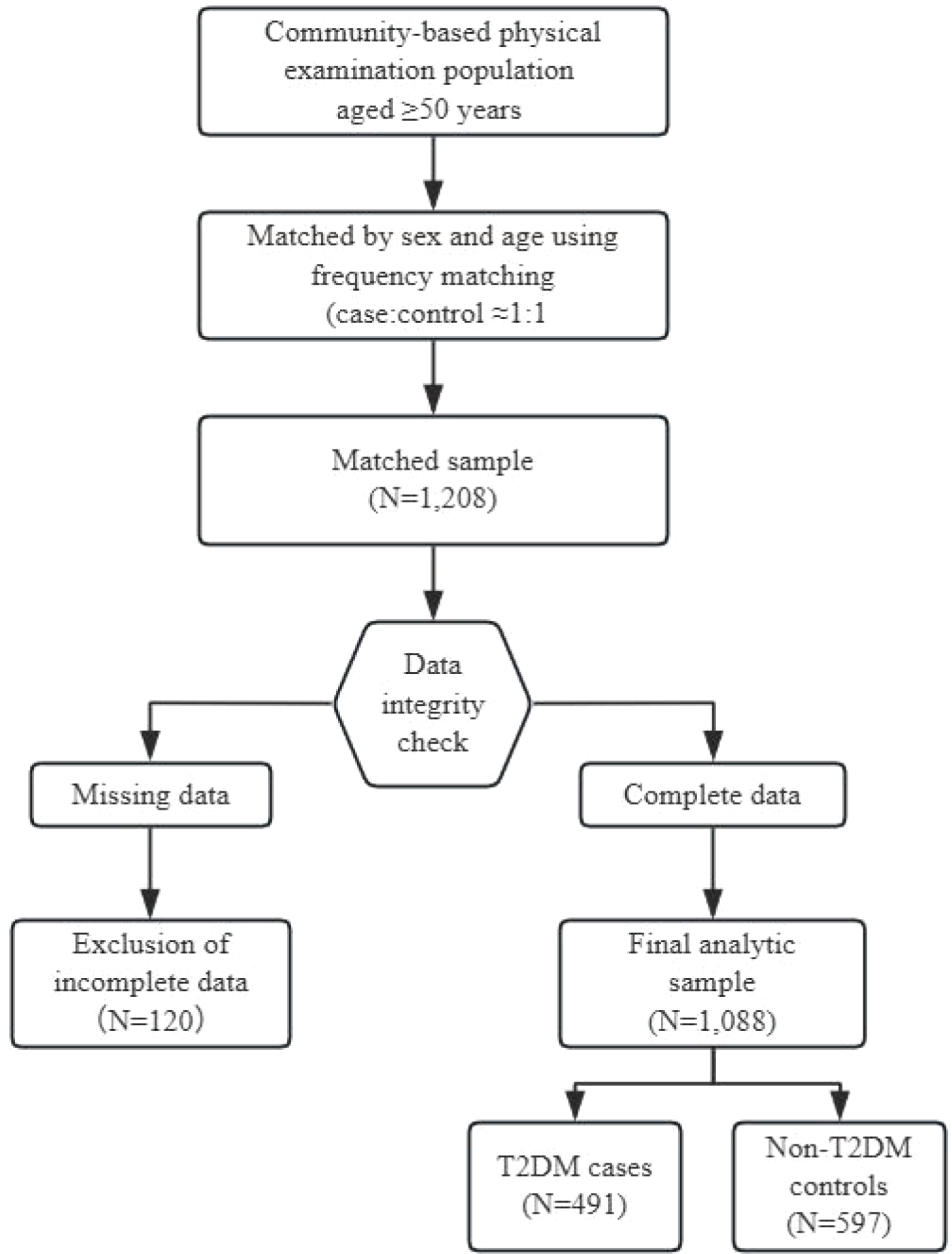
Flow diagram of participant selection.

After matching, 1, 208 participants were included. Among these, 120 individuals were excluded due to incomplete data for calculating the obesity indices (CVAI, LAP, CI, AVI), and complete case analysis was conducted without imputation.The final analytic sample comprised 1, 088 participants (491 T2DM cases and 597 controls), exceeding the sample size requirement of approximately 300 per group estimated for detecting an odds ratio (OR) of 1.5 (highest vs. lowest quartile) with 80% power at α=0.05, assuming 30% exposure prevalence in controls. A flow diagram of participant selection is presented in **Figure 1**.

Detailed reporting of non-participation and refusal rates was limited because this study was based on routinely collected community health examination data without a dedicated prospective registration system. This is a recognized limitation of retrospective secondary analyses based on routine health data (32), as acknowledged in the discussion section.

### 2.2 Data collection and measurement

Assessments were conducted between 8:00 and 10:00 AM in a private, quiet room with a controlled temperature of 22–25 °C to minimize measurement variability. Blood samples were collected after an overnight fast of ≥8 hours, and all laboratory assays were completed within 4 hours of collection. The one-month data collection window (February 2020) ensured consistent protocols across all community health examination centers.

Demographics and clinical history were collected via standardized questionnaire, including age, sex, marital status, occupation, education level, smoking, and drinking. History of hypertension, stroke, coronary heart disease, and glucose-lowering medication use was self-reported and verified against medical records.

Anthropometric measurements followed a standardized protocol. All investigators completed a standardized training session and followed a uniform protocol. Each anthropometric indicator was measured by a single dedicated observer to minimize inter-observer variability. Assessors were blinded to participants’ diabetes status during all anthropometric measurements. Detailed measurement procedures were as follows: (1) Height: Participants stood barefoot with heels, sacrum, and upper back against a vertical stadiometer. The head was positioned in the Frankfort horizontal plane. The horizontal bar was lowered gently to the top of the head, and height was recorded to the nearest 0.1 cm. (2) Weight: Participants wore light clothing and stood barefoot on the center of a calibrated electronic scale. Weight was recorded to the nearest 0.1 kg after stable readings were obtained. (3) Waist circumference (WC): Participants stood upright with feet 30–40 cm apart. WC was measured at the midpoint between the lower costal margin and the iliac crest at the end of normal expiration, to the nearest 0.1 cm. (4) Hip circumference (HC): Measured at the maximum protrusion of the buttocks to the nearest 0.1 cm. All measurements were performed twice, and the average was recorded. If the difference exceeded 1.0 cm, a third measurement was taken, and the average of the two closest values was used. Instruments were calibrated daily.

Index calculations used established scientific formulas, with results standardized to a consistent number of decimal places. Each index was calculated twice independently by two researchers. Discrepancies were resolved by a third senior researcher. A random 10% of participants were selected for manual recalculation to verify consistency. All formulas were reviewed and confirmed by a senior author before formal analysis. All calculations were performed using a consistent procedure and double-checked to avoid data entry errors. The formulas used for calculations are as follows (20, 33, 34):(1) BMI = weight (kg)/height^2^(m^2^);(2) WHR=WC(cm)/HC(cm); (3) WHtR= WC(cm)/height(cm); (4) CVAI (males) = −267.93 + 0.68 × age (year) + 0.03 × BMI (kg/m^2^) + 4.00 × WC (cm) + 22.00 × lg (TG) (mmol/L) − 16.32 × HDL-C (mmol/L); CVAI (females) = −187.32 + 1.71 × age (year) + 4.23 × BMI (kg/m^2^)+ 1.12 × WC (cm) + 39.76 × lg (TG) (mmol/L) − 11.66 × HDL-C (mmol/L); (5) LAP (males) = triglyceride (mmol/L) × [WC (cm)-65]; LAP (females) = triglyceride (mmol/L) × [WC (cm) -58]; (6) CI = WC (m)/0.109 × [weight (kg)/height (m)]^1/2^; (7) AVI = (2 × [WC (cm)]² + 0.7 × [(WC - HC) (cm)]²)/1000.

Biochemical assays were performed on 5 mL fasting venous blood (EDTA tubes), including fasting plasma glucose (FPG), triglyceride (TG), total cholesterol (TC), alanine transaminase (ALT), aspartate transaminase (AST), low-density lipoprotein cholesterol (LDL-C), and high-density lipoprotein cholesterol (HDL-C) using standardized laboratory protocols.

### 2.3 Quality control

All investigators completed standardized training prior to data collection. Daily instrument calibration and random on-site checks were conducted to ensure procedural compliance. All completed questionnaires were double-checked for completeness and logical consistency. Data were independently double-entered to minimize entry errors. Statistical analyses were performed by a professional biostatistician following a pre-specified plan.

### 2.4 Statistical analyses

Analyses were performed using Stata 25.0 and GraphPad Prism 6. Continuous variables are represented by mean ± standard deviation (SD) or median (*P*_25_, *P*_75_). Categorical variables are presented as frequencies and percentages. Chi square (*χ^2^*) test was used to compare categorical variables between the T2DM and non-T2DM groups. The independent t-test or Mann-Whitney U test were used to compare continuous variables between groups.

We divided each adiposity index into quartiles based on the distribution in the total study population. Before logistic regression, statistical assumptions were checked. Multicollinearity was assessed using variance inflation factors (VIF); all VIF values were below 3.0, indicating no significant multicollinearity. The Box-Tidwell test detected no violation of the linearity of the logit for continuous variables, with all *P* > 0.05. Independence of errors was ensured by the study design, as each participant contributed a single observation with no clustering or repeated measures. Model calibration was checked using the Hosmer-Lemeshow goodness-of-fit test (*P* > 0.05), confirming adequate model fit.

To examine associations between each obesity index and prevalent T2DM, we constructed three unconditional logistic regression models with increasing levels of adjustment. Because frequency matching was performed in broad age bands rather than individually, unconditional logistic regression with adjustment for matching factors is appropriate. Model 1(crude) had no adjustment. Model 2 (partially adjusted) adjusted for demographic and lifestyle factors, including age, sex, occupation, marital status, and drinking. Model 3 (fully adjusted) additionally incorporated biochemical indicators (ALT, AST, TC, LDL-C) and comorbidities (hypertension, stroke, and coronary heart disease). Covariates were selected *a priori* based on their established clinical relevance as potential confounders in the adiposity–T2DM association (35). Model 3 served as the primary analytical model for inference. Crude and partially adjusted results are provided in **Supplementary Table 1**.

To evaluate the discriminatory ability of each index for prevalent T2DM within the study sample, receiver operating characteristic (ROC) curve analysis was performed. AUC estimates reflect discriminatory ability within this case-control sample and should not be interpreted as measures of clinical prediction or screening utility.

Sex-stratified logistic regression and ROC analyses were conducted to test whether sex modifies associations and discriminatory ability. Exploratory stratified logistic regression was also performed by hypertension, coronary heart disease, stroke, and BMI to assess the robustness of findings across clinically relevant subgroups (35–38). A two-tailed *P*-value <0.05 was considered statistically significant.

## 3 Results

### 3.1 Baseline characteristics

This study included 1, 088 participants (491 T2DM cases, 597 controls). Age and sex were balanced via frequency matching (**Table 1**). Region, education, religious belief, and smoking showed no between-group differences. T2DM cases had higher proportions of manual workers, married individuals, and comorbidities (hypertension, stroke, coronary heart disease), alongside fewer current drinkers. Biochemical differences were consistent with metabolic dysfunction patterns in T2DM. All obesity indices were significantly higher in T2DM cases (all *P* < 0.001).

**Table 1:** Comparison of baseline characteristics between T2DM and non-T2DM group.

Variables	Total (n=1088)	Non-T2DM (n=597)	T2DM (n=491)	t/*χ^2^*/Z	*P*
Age(years)	70.80 ± 5.91	70.88 ± 5.82	70.70 ± 6.03	0.496	0.620
Gender, n (%)				0.007	0.934
Male	486(44.67)	266(44.56)	220(44.81)		
Female	602(55.33)	331(55.44)	271(55.19)		
Region, n (%)				1.288	0.256
Town	377(34.65)	198(33.17)	179(36.46)		
Countryside	711(65.35)	399(66.83)	312(63.54)		
Occupation, n (%)				35.207	<0.001
Brain-workers	128(11.76)	77(12.90)	51(10.49)		
Manual-workers	733(67.37)	435(72.86)	298(60.69)		
Other	227(20.87)	85(14.24)	142(28.92)		
Educational level, n (%)				4.214	0.122
Illiteracy	533(48.99)	292(48.91)	241(49.08)		
Primary School	282(25.92)	167(27.97)	115(23.42)		
Junior secondary or above	273(25.09)	138(23.12)	135(27.50)		
Marital status, n (%)				4.148	0.042
Be married	863(79.32)	460(77.05)	403(82.08)		
Other	225(20.68)	137(22.95)	88(17.92)		
Religious belief, n (%)				0.101	0.750
No	926(85.11)	505(84.59)	421(85.74)		
Yes	152(13.97)	85(15.41)	67(14.26)		
drinking, n (%)				9.367	0.002
No	723(66.45)	373(62.48)	350(71.28)		
Yes	365(33.55)	224(37.52)	141(28.72)		
Smoking, n (%)				0.000	0.992
No	884(81.25)	485(81.24)	399(81.26)		
Yes	204(18.75)	112(18.76)	92(18.74)		
Hypertension, n (%)				8.063	0.005
No	308(28.31)	190(31.83)	118(24.03)		
Yes	780(71.69)	407(68.17)	373(75.97)		
Stroke, n (%)				8.027	0.005
No	993(91.27)	558(93.47)	435(88.59)		
Yes	95(8.73)	39(6.53)	56(11.41)		
Coronary heart disease, n (%)				24.860	<0.001
No	915(84.10)	532(89.11)	383(78.00)		
Yes	173(15.90)	65(10.89)	108(22.00)		
TC	5.68 ± 1.46	5.93 ± 1.52	5.34 ± 1.33	6.568	<0.001
TG	1.50(1.05, 2.14)	1.33(1.00, 1.86)	1.67(1.19, 2.45)	6.358	<0.001
LDL-C	3.00(2.34, 3.59)	3.08(2.45, 3.65)	2.89(2.22, 3.54)	3.192	0.001
HDL-C	1.72 ± 1.41	1.78 ± 0.55	1.65 ± 1.59	1.666	0.096
ALT	21.00(16.00, 28.35)	20.00(15.60, 27.00)	22.00(16.56, 31.00)	2.852	0.004
AST	26.00(21.50, 32.63)	27.30(23.00, 34.00)	24.50(20.25, 30.00)	5.793	<0.001
BMI, kg/m^2^	24.42 ± 3.62	23.85 ± 3.51	25.12 ± 3.63	5.849	<0.001
WC, cm	87.17 ± 10.95	85.14 ± 10.94	89.64 ± 10.45	6.880	<0.001
HC, cm	94.06 ± 7.20	93.12 ± 7.13	95.13 ± 7.16	4.456	<0.001
WHR	0.92 ± 0.07	0.91 ± 0.07	0.94 ± 0.07	6.676	<0.001
WHtR	0.56 ± 0.07	0.54 ± 0.07	0.57 ± 0.07	5.724	<0.001
CI	1.29 ± 0.09	1.28 ± 0.10	1.31 ± 0.08	5.339	<0.001
AVI	15.44 ± 3.80	14.74 ± 3.70	16.29 ± 3.74	6.840	<0.001
LAP	39.76(20.83, 66.63)	33.60(16.85, 56.67)	48.51(27.50, 79.97)	7.537	<0.001
CVAI	116.86 ± 45.69	108.40 ± 43.07	127.14 ± 46.71	6.874	<0.001

TG, triglyceride; TC, total cholesterol; LDL-C, low-density lipoprotein cholesterol; HDL-C, high-density lipoprotein cholesterol; ALT, Alanine transaminase; AST, aspartate transaminase; BMI, body mass index; WC, waist circumference; HC, hip circumference; WHR, waist to hip ratio; WHtR, waist to height ratio; CVAI, Chinese visceral adiposity index; LAP, lipid accumulation product; CI, conicity index; AVI, abdominal volume index.

### 3.2 Associations between adiposity indices and prevalent T2DM

**Figure 2:**
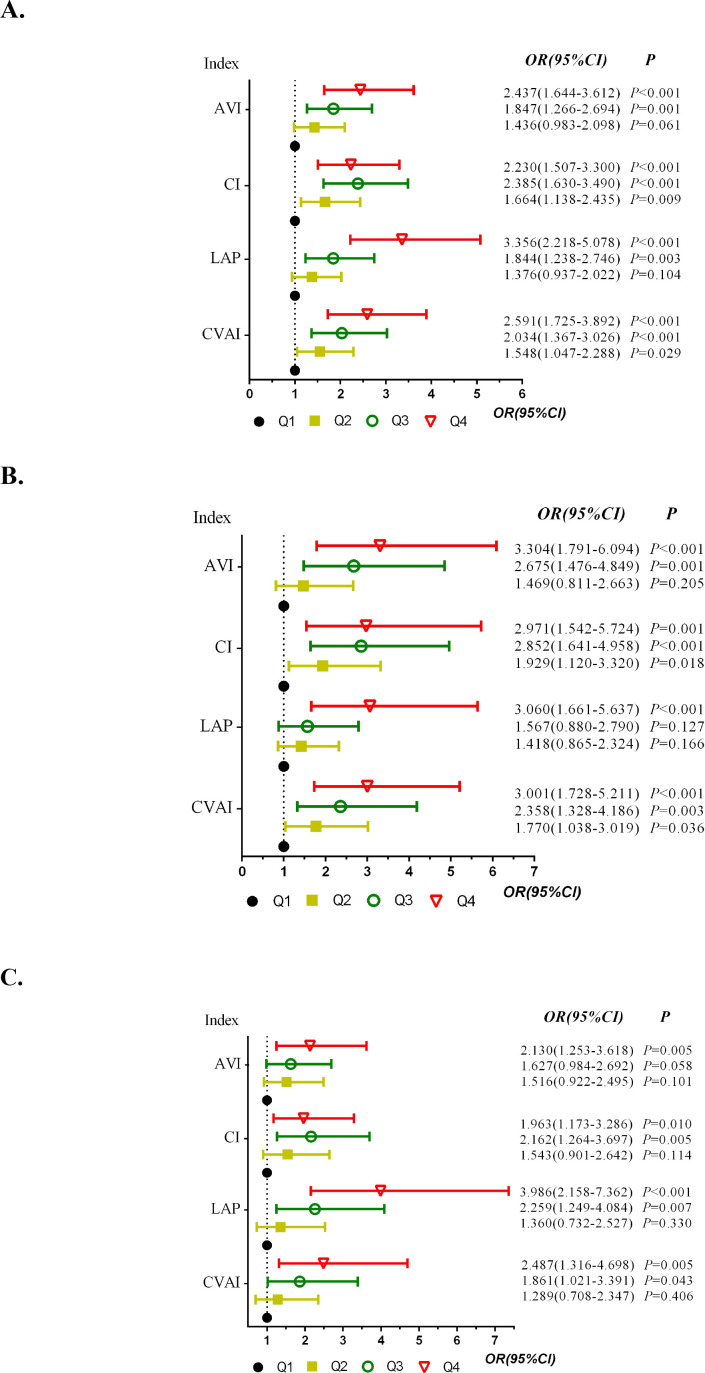
Analysis of the association between CVAI, LAP, CI, AVI and T2DM in Chinese adults aged ≥50 years [**(A)** total population; **(B)** male; **(C)** female].

In the fully adjusted model (Model 3), all four indices were positively associated with prevalent T2DM. The association patterns differed across quartiles: CVAI and CI showed significant positive associations across Q2-Q4 compared to Q1, whereas LAP and AVI were significant only in Q3-Q4 (**Table 2**, **Figure 2A**). The magnitude of associations was attenuated but remained significant after sequential adjustment (**Supplementary Table 1**).

**Table 2:** Logistic regression analysis of the relationship between CVAI, LAP, CI, and AVI and T2DM in Chinese adults aged ≥50 years.

Obesity indices	Group	Model 3 (fully adjusted)	
		*OR(95%CI)*	*P*
CVAI	Q1 (<91.50)	Ref	
	Q2 (91.50-121.68)	1.548(1.047-2.288)	0.029
	Q3 (121.68-147.74)	2.034(1.367-3.026)	<0.001
	Q4 (≥147.74)	2.591(1.725-3.892)	<0.001
LAP	Q1 (<20.85)	Ref	
	Q2 (20.85-39.76)	1.376(0.937-2.022)	0.104
	Q3 (39.76-66.62)	1.844(1.238-2.746)	0.003
	Q4 (≥66.62)	3.356(2.218-5.078)	<0.001
CI	Q1 (<1.23)	Ref	
	Q2 (1.23-1.30)	1.664(1.138-2.435)	0.009
	Q3 (1.30-1.35)	2.385(1.630-3.490)	<0.001
	Q4 (≥1.35)	2.230(1.507-3.300)	<0.001
AVI	Q1 (<12.80)	Ref	
	Q2 (12.80-15.31)	1.436(0.983-2.098)	0.061
	Q3 (15.32-18.05)	1.847(1.266-2.694)	0.001
	Q4 (≥18.05)	2.437(1.644-3.612)	<0.001

Model 3 (fully adjusted): Adjusted for age, sex, occupation, marital status, drinking, ALT, AST, TC, LDL-C, history of hypertension, stroke, and coronary heart disease.

### 3.3 Discriminatory ability for prevalent T2DM within the study sample

**Figure 3:**
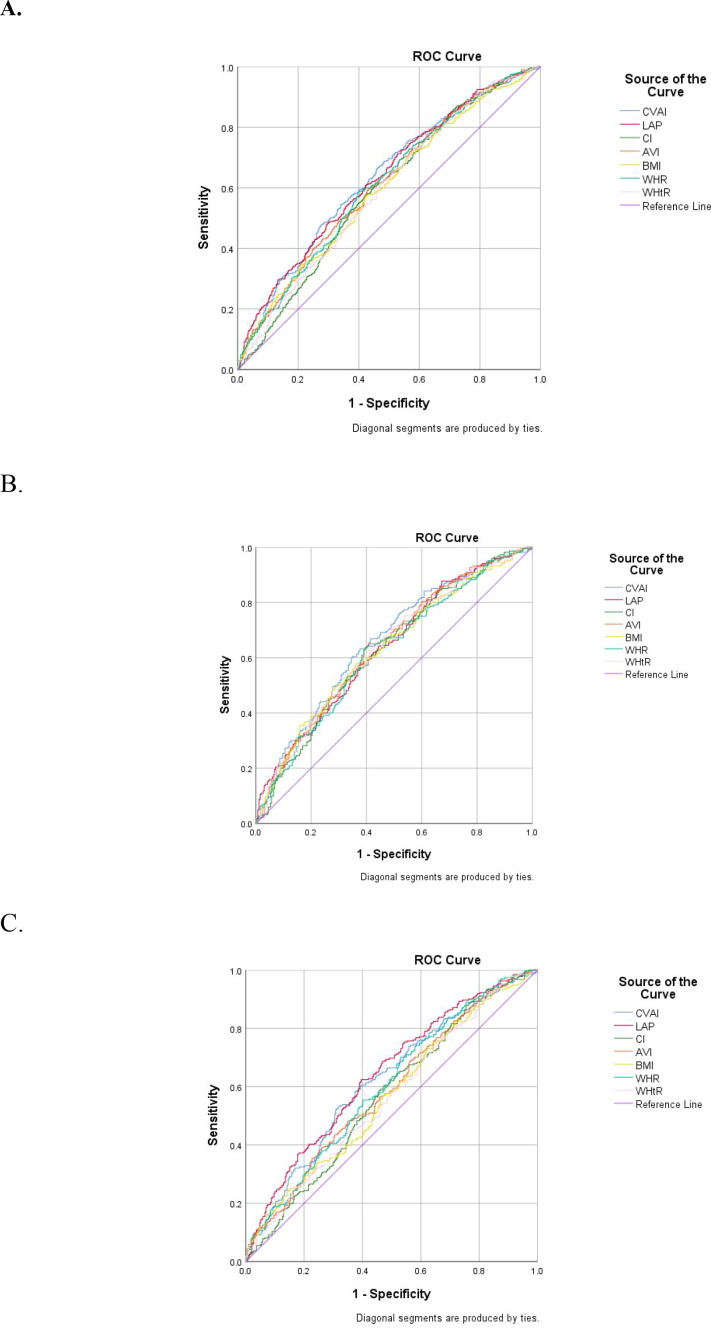
ROC curves for discriminatory ability of adiposity indices for prevalent T2DM within the study sample [**(A)** total population; **(B)** male; **(C)** female].

All indices showed significant discriminatory ability within the study sample (all AUCs > 0.5, *P* < 0.05). CVAI had the numerically highest AUC in the overall population (0.634), followed by LAP (0.633), AVI (0.612), and CI (0.591) (**Table 3**, **Figure 3A**).

**Table 3:** The ROC curve analysis of the relationship between CVAI, LAP, CI, and AVI and T2DM in Chinese adults aged ≥50 years.

Population	Index	AUC (95%CI)	*P*	Cutoff	Sensitivity (%)	Specificity (%)
Total	CVAI	0.634(0.601-0.667)	<0.001	116.46	66.4	54.1
	LAP	0.633(0.600-0.666)	<0.001	51.67	47.9	70.7
	CI	0.591(0.558-0.625)	<0.001	1.30	57.4	58.1
	AVI	0.612(0.579-0.645)	<0.001	15.24	59.7	56.8
Male	CVAI	0.658(0.610-0.707)	<0.001	109.98	63.2	62.4
	LAP	0.636(0.587-0.685)	<0.001	13.66	87.7	32.7
	CI	0.627(0.578-0.676)	<0.001	1.30	53.2	66.9
	AVI	0.642(0.593-0.690)	<0.001	15.07	65.0	59.4
Female	CVAI	0.621(0.577-0.666)	<0.001	134.79	53.1	68.3
	LAP	0.642(0.598-0.686)	<0.001	50.66	62.4	60.4
	CI	0.568(0.522-0.613)	0.004	1.31	58.7	54.4
	AVI	0.586(0.541-0.632)	<0.001	16.20	48.3	65.0

### 3.4 Sex-stratified analyses

Sex-stratified analysis revealed heterogeneity (**Table 4**; **Figures 2B, C**). In males, positive associations were stronger and more consistent for CVAI, CI, and AVI. In females, the association was strongest for LAP. This sex-specific pattern was consistent across adjustment levels.

**Table 4:** Sex-stratified associations of CVAI, LAP, CI, and AVI with T2DM in Chinese adults aged ≥50 years (fully adjusted model).

Obesity indices	Group	Male		Female	
		*OR(95%CI)*	*P*	*OR(95%CI)*	*P*
CVAI	Q1	Ref		Ref	
	Q2	1.770(1.038-3.019)	0.036	1.289(0.708-2.347)	0.406
	Q3	2.358(1.328-4.186)	0.003	1.861(1.021-3.391)	0.043
	Q4	3.001(1.728-5.211)	<0.001	2.487(1.316-4.698)	0.005
LAP	Q1	Ref		Ref	
	Q2	1.418(0.865-2.324)	0.166	1.360(0.732-2.527)	0.330
	Q3	1.567(0.880-2.790)	0.127	2.259(1.249-4.084)	0.007
	Q4	3.060(1.661-5.637)	<0.001	3.986(2.158-7.362)	<0.001
CI	Q1	Ref		Ref	
	Q2	1.929(1.120-3.320)	0.018	1.543(0.901-2.642)	0.114
	Q3	2.852(1.641-4.958)	<0.001	2.162(1.264-3.697)	0.005
	Q4	2.971(1.542-5.724)	0.001	1.963(1.173-3.286)	0.010
AVI	Q1	Ref		Ref	
	Q2	1.469(0.811-2.663)	0.205	1.516(0.922-2.495)	0.101
	Q3	2.675(1.476-4.849)	0.001	1.627(0.984-2.692)	0.058
	Q4	3.304(1.791-6.094)	<0.001	2.130(1.253-3.618)	0.005

Fully adjusted model: Adjusted for age, occupation, marital status, drinking, ALT, AST, TC, LDL-C, history of hypertension, stroke, and coronary heart disease.

Sex-stratified ROC analysis showed a similar pattern (**Table 3**; **Figures 3B, C**). CVAI had the numerically highest AUC in males (0.658), and LAP had the numerically highest AUC in females (0.642).

### 3.5 Exploratory subgroup analyses

Subgroup analyses were performed across hypertension, coronary heart disease, stroke and BMI (**Supplementary Table 2**). Associations remained stable among individuals with or without hypertension and those without coronary heart disease. No significant associations were found in the small stroke subgroup (n=95), likely due to limited statistical power. Associations were stronger in participants with BMI < 24 kg/m² than in those with BMI ≥ 24 kg/m².

## 4 Discussion

This matched case-control study compared four visceral adiposity indices—CVAI, LAP, CI, and AVI—for their associations with prevalent T2DM and discriminatory ability within the study sample, with sex-stratified analyses. The main findings were as follows. First, all four indices were independently associated with prevalent T2DM after full adjustment, but with differing quartile-level sensitivities: CVAI and CI showed significant associations from Q2 onward, whereas LAP and AVI were significant only in Q3-Q4. Second, CVAI had the numerically highest overall discriminatory ability within this sample. Third, sex substantially modified both associations and discriminatory ability, with CVAI, CI, and AVI showing stronger patterns in males and LAP in females.

Visceral adiposity is recognized as a key risk factor for T2DM (39–44). CVAI and CI were positively associated with prevalent T2DM in our study, consistent with prior Chinese studies (26, 45, 46). By contrast, an Italian cross-sectional study found no association between CI and T2DM (30). Several factors may contribute to this discrepancy. The Italian study included adults as young as 18 years, whereas our sample was restricted to those aged ≥50 years. The metabolic consequences of visceral adiposity may be more pronounced in older adults due to age-related sarcopenia and fat redistribution (47). Additionally, for a given BMI, Asian individuals tend to have a higher percent body fat and greater visceral adiposity than Europeans (48, 49), potentially making indices like CI more sensitive in Asian samples. Differences in covariate adjustment may also contribute. The link between LAP and T2DM aligns with evidence from other Asian cohorts (43–45). A systematic review demonstrated that Asian populations, especially Chinese, show a greater risk increase per unit increase in visceral adiposity compared with European populations (41). AVI was first proposed in 2003 to estimate total abdominal volume and explore its relationship with T2DM (50, 51). A 16-year longitudinal study in China identified three AVI trajectories, with faster increase groups showing substantially higher diabetes risks (52).

We observed a distinct pattern in the association between quartiles of each index and T2DM. CVAI and CI exhibited significant positive associations across Q2-Q4, whereas LAP and AVI were significant only in Q3-Q4. This pattern may indicate differing sensitivities across the adiposity spectrum, but the cross-sectional nature of our data precludes definitive conclusions about threshold effects. The more pronounced associations in participants with BMI < 24 kg/m² suggest that these indices may be associated with the “metabolically obese normal-weight” phenotype.

Sex-stratified analyses revealed divergent patterns. CVAI, CI, and AVI showed stronger associations in males, whereas LAP showed a stronger association in females. Several factors likely contribute to these differences. Androgens promote intra-abdominal fat deposition in males (53–55), and the lipid parameters in CVAI capture the dyslipidemic consequences of this visceral fat distribution. CI reflects deviation from a perfect cylindrical body shape. Age-related loss of muscle mass combined with increased abdominal fat in males contributes to a more pronounced “apple-shaped” silhouette that CI captures (56, 57). AVI emphasizes abdominal volume, which increases with intra-abdominal fat accumulation. In contrast, postmenopausal estrogen decline in females leads to increased triglyceride levels and central fat redistribution (58), potentially making LAP—which incorporates triglycerides—more relevant. This sex-specific observation of LAP has been documented in other Chinese cohorts (59–61). Notably, all participants in the present study were aged ≥50 years, and thus caution is warranted when extrapolating the current findings to other age groups.

## 5 Study limitations

Several limitations should be considered when interpreting these findings. The case-control design captures associations between prevalent disease and current adiposity but cannot establish causality. Reverse causality remains plausible. The ROC analysis presented here reflects discriminatory ability within this case-control sample and should not be interpreted as estimates of screening or predictive performance in other settings. Participants were from a single Chinese province, limiting generalizability. Despite comprehensive adjustment, unmeasured confounders (e.g., detailed diet, physical activity, medication use) could influence the observed associations. T2DM was defined by a single fasting glucose measurement, potentially missing isolated postprandial hyperglycemia. Such non-differential misclassification would likely bias results toward the null. Analyses in small subgroups (e.g., the stroke subgroup) were under powered, making negative findings inconclusive. Non-participation and refusal rates could not be reported because this study used a secondary data source.

## 6 Implications

Given the case-control design and single-province origin, these findings are exploratory and generate hypotheses for subsequent testing. The observed sex-specific patterns warrant evaluation in longitudinal cohorts to assess whether these indices are associated with incident T2DM and whether sex-differential patterns for prevalent disease are observed for incident disease. Future studies should standardize biochemical protocols, validate cutoffs in independent samples, and assess performance in medicated populations (e.g., statin users). Prospective replication in independent cohorts is warranted.

## 7 Conclusions

This study compared four visceral adiposity indices—CVAI, LAP, CI, and AVI—for their associations with prevalent T2DM and discriminatory ability within the study sample, with sex-stratified analyses. All indices were independently associated with prevalent T2DM after full adjustment, with differing quartile-level sensitivities. CVAI had the numerically highest discriminatory ability within this sample. Sex substantially modified both associations and discriminatory ability, with CVAI, CI, and AVI showing stronger patterns in males and LAP in females. As a case-control study from a single province, these findings do not establish causality or predictive utility, but generate the testable hypothesis that the discriminatory ability of adiposity indices may differ by sex—pending prospective validation.

## Data Availability

The datasets presented in this study can be found in online repositories. The names of the repository/repositories and accession number(s) can be found in the article/**Supplementary Material**.
