# Supplementary material for: Comparison of the association and discriminatory ability of CVAI, LAP, CI, and AVI for type 2 diabetes in Chinese adults aged ≥ 50 years: a sex-specific analysis
Source: Front Endocrinol (Lausanne). 2026 May 20;17:1829222. doi: 10.3389/fendo.2026.1829222 (PMC13229686; doi:10.3389/fendo.2026.1829222)
Supplement: Supplementary file 1 [file Table1.docx]

Table S1 Logistic regression analysis of the relationship between CVAI, LAP, CI, and AVI and T2DM in Chinese adults aged ≥50 years

| Obesity Indices | Group | Model 1 (crude) | |  | Model 2 (partially adjusted) | |
| --- | --- | --- | --- | --- | --- | --- |
|  |  | *OR(95%CI)* | *P* |  | *OR(95%CI)* | *P* |
| CVAI | Q1 (<91.50) |  |  |  |  |  |
|  | Q2 (91.50-121.68) | 1.643(1.154-2.339) | 0.006 |  | 1.813(1.252-2.627) | 0.002 |
|  | Q3 (121.68-147.74) | 2.277(1.603-3.234) | <0.001 |  | 2.538(1.744-3.694) | <0.001 |
|  | Q4 (≥147.74) | 3.156(2.217-4.492) | <0.001 |  | 4.050(2.568-5.429) | <0.001 |
| LAP | Q1 (<20.85) |  |  |  |  |  |
|  | Q2 (20.85-39.76) | 1.491(1.049-2.120) | 0.026 |  | 1.516(1.055-2.178) | 0.024 |
|  | Q3 (39.76-66.62) | 1.980(1.396-2.807) | <0.001 |  | 2.226(1.535-3.228) | <0.001 |
|  | Q4 (≥66.62) | 3.284(2.308-4.673) | <0.001 |  | 4.050(2.767-5.926) | <0.001 |
| CI | Q1 (<1.23) |  |  |  |  |  |
|  | Q2 (1.23-1.30) | 1.654(1.166-2.346) | 0.005 |  | 1.688(1.181-2.414) | 0.004 |
|  | Q3 (1.30-1.35) | 2.392(1.688-3.390) | <0.001 |  | 2.563(1.793-3.663) | <0.001 |
|  | Q4 (≥1.35) | 2.289(1.615-3.243) | <0.001 |  | 2.524(1.755-3.632) | <0.001 |
| AVI | Q1 (<12.80) |  |  |  |  |  |
|  | Q2 (12.80-15.31) | 1.661(1.169-2.359) | 0.005 |  | 1.695(1.184-2.427) | 0.004 |
|  | Q3 (15.32-18.05) | 2.168(1.529-3.074) | <0.001 |  | 2.179(1.523-3.117) | <0.001 |
|  | Q4 (≥18.05) | 2.877(2.024-4.089) | <0.001 |  | 3.187(2.218-4.578) | <0.001 |

Model 1(crude): Adjusted for none; Model 2 (partially adjusted): Adjusted for age, gender, occupation, marital status, and drinking

Table S2 Stratified analysis of the association between CVAI, LAP, CI, and AVI and T2DM in Chinese adults aged ≥50 years

|  | Group | CVAI | |  | LAP | |  | CI | |  | AVI | |
| --- | --- | --- | --- | --- | --- | --- | --- | --- | --- | --- | --- | --- |
|  |  | OR (95%CI) | *P* |  | OR (95%CI) | *P* |  | OR (95%CI) | *P* |  | OR (95%CI) | *P* |
| Hypertension |  |  |  |  |  |  |  |  |  |  |  |  |
| Yes | Q1 | Ref |  |  | Ref |  |  | Ref |  |  | Ref |  |
|  | Q2 | 1.822 (1.121-2.963) | 0.016 |  | 1.412(0.880-2.266) | 0.153 |  | 1.349(0.841-2.164) | 0.214 |  | 1.141(0.711-1.830) | 0.584 |
|  | Q3 | 2.211(1.343-3.639) | 0.002 |  | 1.505(0.925-2.449) | 0.100 |  | 1.819(1.143-2.892) | 0.012 |  | 1.738(1.082-2.793) | 0.022 |
|  | Q4 | 2.288(1.398-3.743) | 0.001 |  | 2.458(1.490-4.053) | <0.001 |  | 1.888(1.181-3.019) | 0.008 |  | 1.891(1.183-3.024) | 0.008 |
| No | Q1 | Ref |  |  | Ref |  |  | Ref |  |  | Ref |  |
|  | Q2 | 1.049(0.522-2.108) | 0.893 |  | 1.138(0.570-2.271) | 0.715 |  | 2.309(1.196-4.455) | 0.013 |  | 2.275(1.175-4.403) | 0.015 |
|  | Q3 | 1.832(0.915-3.669) | 0.087 |  | 2.919(1.410-6.043) | 0.004 |  | 4.969(2.451-10.075) | <0.001 |  | 2.047(1.066-3.932) | 0.031 |
|  | Q4 | 7.245(2.980-17.614) | <0.001 |  | 10.371(4.256-25.272) | <0.001 |  | 2.685(1.219-5.914) | 0.014 |  | 6.224(2.614-14.822) | <0.001 |
| Coronary heart disease | |  |  |  |  |  |  |  |  |  |  |  |
| Yes | Q1 | Ref |  |  | Ref |  |  | Ref |  |  | Ref |  |
|  | Q2 | 1.318(0.453-3.830) | 0.612 |  | 2.125(0.731-6.180) | 0.166 |  | 2.799(0.963-8.136) | 0.059 |  | 4.419(1.532-12.748) | 0.006 |
|  | Q3 | 2.333(0.733-7.427) | 0.151 |  | 5.642(1.725-18.457) | 0.004 |  | 4.426(1.545-12.681) | 0.006 |  | 7.013(2.193-23.006) | 0.001 |
|  | Q4 | 2.181(0.744-6.390) | 0.155 |  | 6.677(1.956-22.792) | 0.002 |  | 3.321(1.167-9.446) | 0.024 |  | 3.891(1.372-11.038) | 0.011 |
| No | Q1 | Ref |  |  | Ref |  |  | Ref |  |  | Ref |  |
|  | Q2 | 1.645(1.080-2.506) | 0.021 |  | 1.274(0.844-1.923) | 0.248 |  | 1.540(1.024-2.317) | 0.038 |  | 1.278(0.848-1.927) | 0.241 |
|  | Q3 | 2.071(1.348-3.181) | 0.001 |  | 1.633(1.064-2.506) | 0.025 |  | 2.181(1.444-3.292) | <0.001 |  | 1.591(1.060-2.386) | 0.025 |
|  | Q4 | 2.831(1.808-4.431) | <0.001 |  | 3.108(1.978-4.881) | <0.001 |  | 2.177(1.418-3.344) | <0.001 |  | 2.466(1.601-3.798) | <0.001 |
| Stroke |  |  |  |  |  |  |  |  |  |  |  |  |
| Yes | Q1 | Ref |  |  | Ref |  |  | Ref |  |  | Ref |  |
|  | Q2 | 5.716(0.999-32.712) | 0.050 |  | 0.656(0.119-3.609) | 0.628 |  | 0.848(0.145-4.960) | 0.855 |  | 2.003(0.395-10.159) | 0.402 |
|  | Q3 | 3.306(0.483-22.626) | 0.223 |  | 1.380(0.212-9.001) | 0.736 |  | 1.567(0.290-8.463) | 0.602 |  | 2.006(0.387-10.398) | 0.407 |
|  | Q4 | 4.515(0.684-29.806) | 0.117 |  | 1.597(0.882-2.892) | 0.204 |  | 0.910(0.169-4.907) | 0.913 |  | 1.435(0.273-7.549) | 0.670 |
| No | Q1 | Ref |  |  | Ref |  |  | Ref |  |  | Ref |  |
|  | Q2 | 1.453(0.967-2.183) | 0.072 |  | 1.385(0.932-2.059) | 0.107 |  | 1.762(1.185-2.620) | 0.005 |  | 1.432(0.964-2.128) | 0.076 |
|  | Q3 | 2.111(1.397-3.191) | <0.001 |  | 1.989(1.316-3.006) | 0.001 |  | 2.673(1.791-3.989) | <0.001 |  | 1.931(1.300-2.869) | 0.001 |
|  | Q4 | 2.753(1.797-4.217) | <0.001 |  | 3.460(2.231-5.366) | <0.001 |  | 2.569(1.694-3.895) | <0.001 |  | 2.786(1.840-4.218) | <0.001 |
| BMI |  |  |  |  |  |  |  |  |  |  |  |  |
| <24 | Q1 | Ref |  |  | Ref |  |  | Ref |  |  | Ref |  |
|  | Q2 | 1.764(1.095-2.843) | 0.020 |  | 1.354(0.842-2.176) | 0.211 |  | 1.628(1.009-2.624) | 0.046 |  | 1.530(0.995-2.352) | 0.053 |
|  | Q3 | 2.374(1.272-4.433) | 0.007 |  | 2.812(1.533-5.158) | 0.001 |  | 2.809(1.594-4.950) | <0.001 |  | 2.122(1.176-3.828) | 0.012 |
|  | Q4 | 1.157(0.228-5.871) | 0.861 |  | 2.815(1.383-5.732) | 0.004 |  | 1.227(0.663-2.273) | 0.515 |  | 1.464(0.422-5.078) | 0.548 |
| ≥24 | Q1 | Ref |  |  | Ref |  |  | Ref |  |  | Ref |  |
|  | Q2 | 0.948(0.305-2.950) | 0.926 |  | 0.906(0.378-2.171) | 0.825 |  | 1.546(0.745-3.208) | 0.242 |  | 2.171(0.586-8.040) | 0.246 |
|  | Q3 | 1.719(0.571-5.179) | 0.336 |  | 1.084(0.456-2.576) | 0.856 |  | 2.157(1.061-4.383) | 0.034 |  | 3.095(0.867-11.052) | 0.082 |
|  | Q4 | 2.626(0.873-7.901) | 0.086 |  | 2.556(1.065-6.139) | 0.036 |  | 2.787(1.358-5.721) | 0.005 |  | 4.564(1.271-16.381) | 0.020 |

Adjusted (fully adjusted) for age, gender, occupation, marital status, drinking, ALT, AST, TC, LDL-C, history of hypertension, stroke, and coronary heart disease (The subgroups were not adjusted for stratification factors). CVAI: Chinese visceral adiposity index; LAP: lipid accumulation product; CI: conicity index; AVI: abdominal volume index

Table S3 The ROC curve analysis of the relationship between BMI, WHR, and WHtR and T2DM in Chinese adults aged ≥50 years

| Population | Index | AUC (95%CI) | *P* | **Cutoff** | **Sensitivity (%)** | **Specificity (%)** |
| --- | --- | --- | --- | --- | --- | --- |
| Total | BMI | 0.596(0.562-0.630) | <0.001 | 24.35 | 57.4 | 57.5 |
|  | WHR | 0.611(0.578-0.644) | <0.001 | 0.93 | 58.7 | 60.1 |
|  | WHtR | 0.593(0.560-0.627) | <0.001 | 0.53 | 72.9 | 41.9 |
| Male | BMI | 0.631(0.582-0.681) | <0.001 | 24.33 | 58.2 | 63.5 |
|  | WHR | 0.623(0.573-0.672) | <0.001 | 0.93 | 64.1 | 59.8 |
|  | WHtR | 0.639(0.590-0.688) | <0.001 | 0.52 | 70.0 | 51.9 |
| Female | BMI | 0.567(0.521-0.613) | 0.005 | 22.99 | 74.9 | 36.0 |
|  | WHR | 0.601(0.556-0.646) | <0.001 | 0.91 | 70.1 | 45.9 |
|  | WHtR | 0.564(0.518-0.610) | 0.007 | 0.53 | 80.1 | 29.6 |
